# The Relationship Between Workplace Ostracism and Sleep Quality: A Mediated Moderation Model

**DOI:** 10.3389/fpsyg.2019.00319

**Published:** 2019-03-04

**Authors:** Yang Chen, Shuang Li

**Affiliations:** School of Management, China University of Mining and Technology, Xuzhou, China

**Keywords:** workplace ostracism, coping humor, psychological detachment, sleep quality, mediated moderation model

## Abstract

Extant research suggests that workplace ostracism has a detrimental impact on the outcomes of employees. However, very little is known about the impact of workplace ostracism on sleep quality. Therefore, this study aimed to address this gap in the literature. By employing the extended stressor-detachment model, we investigated the mediating role of psychological detachment and the moderating role of coping humor. We used a self-report questionnaire and a time-lagged research design to assess employees’ workplace ostracism, coping humor, psychological detachment, and sleep quality. A total of 403 valid questionnaires were obtained from participants in full-time employment in China. As predicted, the results showed that workplace ostracism is negatively associated with psychological detachment, which in turn, impairs sleep quality. We further found that high levels of coping humor buffer the negative effect of workplace ostracism on psychological detachment and sleep quality. In addition, the moderation effect of coping humor on the relationship between workplace ostracism and sleep quality is mediated by psychological detachment. Finally, based upon the results, we discuss the theoretical implications, provide practical proposals for managers and policymakers, and make suggestions for the direction of further research.

## Introduction

Recovering from stress is essential in upholding individual health and well-being (Geurts and Sonnentag, 2006). Research has shown that individuals experiencing incomplete recovery are vulnerable to serious health threats, such as hypertension (Hocking Schuler and O’Brien, 1997) and even cardiovascular death (Kivimäki et al., 2006). Sleep is one of the most important recovery activities (Barnes et al., 2012), which takes up nearly one-third of each individual’s daily time (Lallukka, 2010). Unfortunately, inadequate sleep is becoming a widespread and serious concern. For example, in a large survey of 35,327 individuals who were mainly in employment, from 10 different countries, 24% reported experiencing poor sleep. Furthermore, 26% of 10,079 Chinese participants have reported suffering from sleep impairment (Soldatos et al., 2005). In another survey conducted in the United States, 26% of a total of 1,000 employees who were working 30 h or more per week reported that they were not sleeping well (Swanson et al., 2011). Low sleep quality often gives rise to serious negative outcomes, including workplace accidents and death (Hublin et al., 2007; Uehli et al., 2014), coronary heart disease (Liu and Tanaka, 2002), mental disorders (Breslau et al., 1996), absence from work due to illness (Niedhammer et al., 2009), low workplace productivity (Rosekind et al., 2010), and poor job performance (Kessler et al., 2011). Although previous studies have explored sleep quality in the context of work stressors and the development of health impairments, the majority of them have focused on shift-work (e.g., Sallinen and Kecklund, 2010; Lin et al., 2014), workload (Åkerstedt et al., 2002; Dorrian et al., 2011) and work-family conflict/balance (Berkman et al., 2010; Lallukka et al., 2014; Buxton et al., 2016). Other important stressors, such as stressful interpersonal relationships at work, have been neglected (Pereira et al., 2013).

Interpersonal relationships are an important factor influencing sleep quality (Kent et al., 2015). Given the significant amount of time that organizational members spend with one another (Chung, 2018), teamwork and cooperation are now emphasized by organizations (Salas et al., 2015). Consequentially, employees cannot avoid interacting with others (i.e., coworkers and supervisors) in the workplace. If individuals experience stressful interpersonal relationships, they will be prone to suffering impaired quality of sleep. As a form of negative and specific social interaction (Ali Khan, 2017), workplace ostracism is a prevalent phenomenon (Ferris et al., 2008b) and anyone can engage in exclusionary behavior, regardless of their role in the workplace (Williams, 2007). Furthermore, as workplace ostracism can affect individuals’ outcomes in non-work domains via a spillover effect (Liu et al., 2013), workplace ostracism may be responsible for poor sleep quality. So far, there is only one empirical study, namely, that by Pereira et al. (2013), that has tested the relationship between social exclusion at work and sleep quality by using worries about work as a mediator variable. The results showed that this relationship was not mediated by worries about work. Therefore, we know little about the mechanisms through which workplace ostracism negatively affects sleep quality. In addition, the boundary conditions of workplace ostracism in influencing sleep quality have received only limited attention. To address these gaps in the literature, the present study aimed to replicate the finding of a relationship between workplace ostracism and sleep quality and to expand the previous literature by examining a potential mediator (i.e., psychological detachment) and moderator (i.e., coping humor) to help explain this relationship from a recovery perspective.

Research on recovery suggests that leaving the work environment physically does not automatically imply that recovery occurs. Rather, if individuals are unable to psychologically detach from work-related stressful events during non-work time, the stress-related psychophysiological activation can be prolonged or reactivated, thereby impairing the recovery processes (e.g., sleep) (Etzion et al., 1998; Sonnentag and Bayer, 2005). In addition, researchers have suggested that specific resources can play the role of buffer for specific job stressors (Bakker and Demerouti, 2007). Coping humor, a form of personal resource (Führ et al., 2015), has a stress-moderating effect (Martin and Dobbin, 1988), and in particular, moderates the stress from social interaction (Nezlek and Derks, 2001). As workplace ostracism is a stressor resulting from social interaction, we tested the moderating effect of coping humor in the relationship between workplace ostracism and psychological detachment.

In the field of recovery, the extended stressor-detachment (ESD) model (Sonnentag and Fritz, 2015) provides a theoretical framework for our study variables. This model emphasizes the role of psychological detachment in the stressor-strain process. The core assumptions of the ESD model are that (a) workplace stressors can elicit negative activation which causes individuals to fail to sufficiently psychologically detach from work during non-work time, which in turn, triggers an impaired recovery process or negative outcomes; (b) some factors (e.g., attentional processes, personal and job resources) may moderate the effects of workplace stressors on psychological detachment. Therefore, based on the ESD model, the current study empirically tested the mediating effect of psychological detachment on the relationship between workplace ostracism and sleep quality. Additionally, the stress-buffering effect of coping humor on the relationship between workplace ostracism and psychological detachment, and sleep quality, was examined.

## Literature Review and Hypotheses

### Workplace Ostracism as Workplace Stressor

Ostracism, one of the “social pains” (Eisenberger et al., 2003), refers to when individuals perceive themselves to be ignored, excluded, and disrespectfully treated by others (Ferris et al., 2008a). Ostracism is a prevalent phenomenon in kinds of important social contexts, including in the workplace (Fox and Stallworth, 2005). In the workplace, an individual may feel isolated or disconnected from his/her colleagues through social interaction, including the avoidance of eye contact, leaving the room when he/she enters, or failing to respond to his/her greetings (Williams, 2007; Robinson et al., 2013; Zhu et al., 2017).

Workplace ostracism comprises three key characteristics. First, workplace ostracism does not require the motivation to cause harm to an individual. For example, it may be that at times a person is too engaged with his/her own work to notice other colleagues. Second, behaviors can be regarded as workplace ostracism when a person (or group) omits to include another colleague when it is socially appropriate to do so. Thus, what an individual perceives ostracism, it may not be considered the same way in another. Third, workplace ostracism refers to paying little positive attention to others, rather than displaying negative attention (Robinson et al., 2013; Zhu et al., 2017).

Workplace ostracism can be regarded as a workplace stressor which can result in stress (Chung, 2018). Employees who are being ostracized are likely to experience negative self-perceptions and negative emotions, which in turn, cause them to experience a lack of control and loss of resources. Under the influence of workplace ostracism, employees may feel their social ties to others are weakened (Jahanzeb and Fatima, 2017; Yang and Treadway, 2018). That is to say, their social supports and their sense of belonging, which have been argued to be critical resources in stressful circumstances (Hobfoll, 1989), are threatened. Accordingly, workplace ostracism is a painful and aversive experience, and ostracized employees are prone to perceive stress once they have suffered from these negative experiences (Chung, 2018). Finally, Williams (2007) suggested that ostracism can be argued to be an interpersonal stressor, therefore, sparking stress. All of factors mentioned above suggest that workplace ostracism can be a workplace stressor.

### Mediating Role of Psychological Detachment

Psychological detachment is defined as when individuals are mentally disengaged from work-related activities, thoughts, ruminations or worries, during non-work time (Sonnentag and Fritz, 2007). It describes a state in which individuals’ physiology and psychology are not at all interfered with by work-related issues during their off-work time (Sonnentag and Bayer, 2005). It should be noted that a lack of psychological detachment overlaps with concepts including worry and rumination (Sonnentag and Fritz, 2015).

Some studies in the field of general ostracism showed that ostracism can significantly increase individuals’ likelihood of rumination (Zwolinski, 2012) and anxiety (Wesselmann et al., 2018). Thus, we propose that perceived workplace ostracism may inhibit psychological detachment. According to the ESD model, we know that workplace stressors prevent individuals from achieving psychological detachment. More specifically, workplace ostracism is a painful and aversive experience (Ferris et al., 2008b) which stimulates employees’ negative activation (e.g., negative affect, worry, and rumination), triggers their recall of this negative experience (i.e., experiences of ostracism at work) during after-work hours, and induces worry about potential reoccurrence the following day. In this case, it is more difficult for employees to mentally disengage from work during non-work time (Sonnentag and Fritz, 2015). In an empirical study on 349 construction workers, Chen and Li (2018) demonstrated that workplace ostracism hampers employees’ psychological detachment. Accordingly, we proposed:

Hypothesis 1: Workplace ostracism is negatively related to psychological detachment.

The ESD model indicates that psychological detachment is significantly related to strain reaction and poor well-being. As an indicator of strain and well-being (Hahn et al., 2011), sleep quality may be positively affected by psychological detachment (Sonnentag et al., 2010; Clinton et al., 2017). One explanation for this is that if individuals are unable to psychologically detach from work at bedtime, their cognitive and affective processes may be adversely activated by negative work-related issues (e.g., negative events and emotions) (Brosschot et al., 2006). These may spark their prolonged activation and lead to difficulties for the individual in falling asleep (Clinton et al., 2017). In addition, existing research has confirmed that an inability to mentally detach from work during an evening is associated with impaired sleep quality (Pereira and Elfering, 2014a). For example, based on a diary study across 42 days, Åkerstedt et al. (2012) found that failing to psychologically detach from work (i.e., ruminating about work) at bedtime reduces sleep quality. Therefore, we proposed that:

Hypothesis 2: Lower psychological detachment predicts lower sleep quality.

Following the ESD model, workplace stressors make it difficult for individuals to detach from work during downtime, which may elicit a strain reaction and poor well-being (Sonnentag and Fritz, 2015). Additionally, based on the preceding Hypotheses (1 and 2), we proposed that when employees perceive workplace ostracism they will ruminate and be unable to unwind from this painful and aversive experience during non-work time, leading to insufficient psychological detachment. This keeps employees in a prolonged or reactivated state of psychophysiological arousal even when they are out of the workplace, then triggering poor sleep quality during the evening (Pereira and Elfering, 2014a). We therefore proposed:

Hypothesis 3: The relationship between workplace ostracism and sleep quality is mediated by psychological detachment.

### The Moderating Effect of Coping Humor

That coping humor is a useful and readily available tool to cope with stress is a popular idea (Martin and Lefcourt, 1983; Sliter et al., 2014). It is a coping strategy frequently adopted by individuals under stressful circumstances (Ziv, 1988), and refers to the humor an individual uses to seek to eliminate the effects of stressors he/she is encountering. In short, it emphasizes that individuals adopt a humorous perspective in the face of difficulties (Martin, 2007; Rieger and Mcgrail, 2013). Existing research suggests that coping humor has a significant stress-buffering effect (Martin, 1996; Chen and Martin, 2007). For example, in their sample of 179 firefighters, Sliter et al. (2014) found that coping humor can buffer the effects of traumatic stressors on post-traumatic stress disorder, burnout, and absenteeism. Moreover, Martin and Dobbin (1988) confirmed that coping humor plays a moderator role in the relationship between daily hassles and secretory immunoglobulin A levels, namely, the relationship between them was weaker when the scores on the coping humor measure were high.

In the present study, we argue that coping humor may moderate the negative effect of workplace ostracism on psychological detachment in the following ways. First, as mentioned above, the ESD model proposes that workplace stressors can trigger negative activation such as negative affect (Sonnentag and Fritz, 2015), job anxiety (Chen et al., 2017), or rumination (Donahue et al., 2012) and this negative activation hinders individuals from being able to mentally detach from work even though he/she has physically left the work situation (Sonnentag and Fritz, 2015). However, some personal resources can attenuate the impact of workplace stressors on psychological detachment (Sonnentag and Fritz, 2015). As a form of personal resource (Führ et al., 2015), coping humor helps individuals to cope with difficult events using a positive cognitive reappraisal (Lefcourt, 2001; Wilkins, 2013) which can decrease negative activation (e.g., anxiety, worry, and rumination) (Führ et al., 2015; Jose et al., 2017). Based on these views and Hypothesis 1, we know that workers perceiving workplace ostracism may engage in a variety of negative emotions (e.g., anxiety and depression) and ruminate about their experiences of ostracism during non-work time, which causes prolonged activation and inhibits mental disengagement from work. In this case, if ostracized individuals use coping humor to cope with workplace ostracism, they will appraise workplace ostracism with positive cognitions and their self-esteem and confidence in their ability to resolve stress are increased (Sliter et al., 2014). This can weaken the negative effect of workplace ostracism, thus avoiding the negative affect, worry, or rumination caused by workplace ostracism, which in turn eliminates negative activation and promotes psychological detachment. Therefore, we proposed:

Hypothesis 4: Coping humor moderates the relation between workplace ostracism and psychological detachment so that the negative relation will be weaker when the scores for coping humor are high.

In addition, coping humor may also buffer the relationship between workplace ostracism and sleep quality. Individuals experiencing unpleasant interpersonal relationships are likely to have difficulty falling asleep during the evening time (Roth and Ancoli-Israel, 1999). As workplace ostracism is an interpersonal stressor (Williams and Sommer, 1997), employees under the influence of workplace ostracism are likely to experience poor sleep quality. Moreover, previous research has suggested that under the influence of workplace ostracism, employees suffer from sleep fragmentation (Pereira et al., 2013). As mentioned above, coping humor can weaken the detrimental effects of workplace ostracism. Therefore, employees with high levels of coping humor are less likely to suffer from poor sleep quality than those with low levels of coping humor when experiencing workplace ostracism. As such, we proposed:

Hypothesis 5: Coping humor moderates the relationship between workplace ostracism and sleep quality so that the negative relationship will be weaker when the scores for coping humor are high.

### A Mediated Moderation Model

We combined the arguments above in our proposed mediated moderation model. We have proposed that coping humor can buffer the effects of workplace ostracism on psychological detachment and sleep quality, and that psychological detachment mediates the relation between workplace ostracism and sleep quality. Therefore, we argue that coping humor can buffer the negative effect of workplace ostracism on psychological detachment, thereby improve sleep quality. Therefore, we hypothesized the following:

Hypothesis 6: The negative moderating effect of coping humor on the relationship between workplace ostracism and sleep quality is mediated by psychological detachment.

**FIGURE 1:**
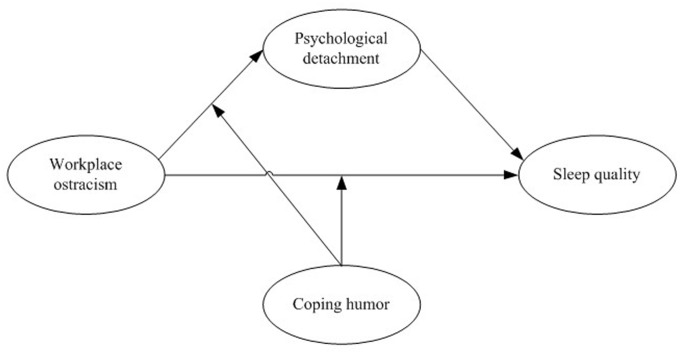
Conceptual model.

Based on the ESD model and previous studies, we built a multivariate model to test the proposed hypotheses. The conceptual model is depicted in Figure 1.

## Materials and Methods

### Participants and Procedures

All the participants were full-time employees from four organizations in three provinces (Shanxi, Jiangsu, and Anhui). These organizations were based in the medical, real estate, and telecommunications industries. To alleviate the effect of common method bias (CMB), our data were collected in two separate stages. However, if the time interval between two stages is inordinately short, the salience of the questionnaire in first stage may interfere with the evaluations of participants in second stage, thereby impairing the causal link among our study variables. If the interval is inordinately long, then it may allow the causal effect to dissipate over time (Podsakoff et al., 2003; Zhao and Xia, 2017). The 1-month time lag was a common practice when data were collected in different stages (e.g., Michel et al., 2016; Naseer et al., 2018). This is because a 1-month time lag is neither too short nor too long, which appears to fit the criteria mentioned above (Dawson et al., 2016). Therefore, we employed a 1-month lag in the present study. In the first stage of the survey (T1), participants were required to provide basic demographic information (i.e., gender, age, marital status, and daily working hours) and completed measures of workplace ostracism and coping humor. One-month after T1, the second stage of the survey (T2) was conducted. At T2, employees completed measures of psychological detachment and sleep quality. This study was approved by the institutional review board of our university.

Before collecting data, we contacted the HR managers of organizations, explained the purpose of the study to them, and invited them to participate. Our research team was assigned to different organizations to collect data after we received permission from the HR managers. Employees were encouraged to participate in our study and were assured that their confidentiality and anonymity would be protected. All participants took part voluntarily and were provided with a unique identification number so we could match their responses at T1 with those at T2. At each stage, participants were allowed to complete the paper-and-pencil questionnaire which took less than 15 min in a meeting room and returned their completed questionnaire to our team members directly.

At T1, we issued a total of 520 questionnaires of which 493 were returned (94.8% response rate). Questionnaires with illegible responses were removed (19 responses). Following these exclusions, 474 valid questionnaires remained (91.2% valid response rate). At T2, the questionnaires were sent to 474 employees whose responses were valid in the first stage survey and 417 were returned, which accounted for 88.0% of 474 (88.0% response rate). After removing some illegible responses from 417, we finally obtained 403 valid questionnaires, which accounted for 77.5% of 520 (77.5% valid response rate). The final sample contained 208 males (51.6%) and 195 females (48.4%). The mean age of participants was 34.03 years old (*SD* = 6.74). The average for the daily working hours of participants was 10.44 (*SD* = 2.42). Regarding marital status, 21.8% of the respondents were unmarried, 74.9% were married, and 3.2% were of other marital status.

### Measures

Self-report questionnaires were used to assess our study variables. Given that the participants were all Chinese, the original English items were translated into Chinese. The double-blind back-translation procedure (Brislin, 1980) was used to create Chinese versions of all instruments. In order to avoid potential inaccuracies as far as possible, we also interviewed several HR managers with rich managerial experience in their respective industries. Cronbach’s alpha was used to measure the internal consistency of each instrument.

#### Workplace Ostracism

We used a 10-item scale adopted from the work of Ferris et al. (2008b) to measure employees’ perceptions of workplace ostracism. The responses of participants for items were 1 (never) to 5 (very often). Sample items include: “Others ignored you at work” and “Others at work treated you as if you weren’t there.” Cronbach’s alpha for this scale was 0.89.

#### Coping Humor

Coping humor was measured by the seven-item scale developed by Martin and Lefcourt (1983). This measurement is used to evaluate the degree to which individuals use humor as a method to handle stressful events. Each item was measured on a five-point scale (1 = strongly disagree, 5 = strongly agree). Example items are “I can usually find something to laugh or joke about even in trying situations” and “It has been my experience that humor is often a very effective way of coping with problems.” Cronbach’s alpha was 0.83.

#### Psychological Detachment

The measurement of psychological detachment was the subscale for psychological detachment adopted from the Recovery Experience Questionnaire (Sonnentag and Fritz, 2007). This subscale includes four items to assess to what extent participants were able to mentally disengage from work during after-work hours. Each item was measured on a five-point scale (1 = completely disagree, 5 = absolutely agree). Sample items include “I don’t think about work at all during non-work time” and “I get a break from the demands of work during non-work time.” Cronbach’s alpha was 0.77.

#### Sleep Quality

Sleep quality was assessed by the four-item scale developed by Bravo et al. (2017). The items evaluated the quality of sleep, sleepiness, satisfaction, and trouble with sleep, respectively, with a five-point scale. Specifically, participants’ ratings for sleepiness and trouble with sleep items ranged from 1 (never) to 5 (quite often), the quality of sleep item ranged from 1 (extremely poor) to 5 (very good), and the sleep satisfaction item ranged from 1 (quite dissatisfied) to 5 (fully satisfied). Sample items are “How would you rate your quality of sleep? (quality of sleep)” and “How often do you feel tired, fatigued or sleepy during the day? (sleepiness).” Cronbach’s alpha for this scale was 0.79.

#### Control Variables

As previous research has identified that participants’ gender, age, marital status, and daily working hours impact on sleep quality (Åkerstedt et al., 2002; Troxel et al., 2010; Hülsheger et al., 2015), they were used as control variables in this study to eliminate their potential effects.

## Results

### Confirmatory Factor Analysis (CFA)

We adopted CFA to evaluate the discriminant and convergent validity of our core study variables: workplace ostracism, coping humor, psychological detachment, and sleep quality. Typically accepted indices were used to determine the model fit. The indices were as follows: χ^2^/*df*, comparative fit index (CFI), goodness-of-fit index (GFI), Tucker-Lewis index (TLI) and root mean square error of approximation (RMSEA). Among them, when the values of CFI, GFI, and TLI are more than 0.9 (Tucker and Lewis, 1973; Salisbury et al., 2002), the value of χ^2^/*df* ranges from 1 to 5 (Salisbury et al., 2002), and the value of RMSEA is below 0.08 (Byrne, 2006), then the model can be accepted. If there were several models that could be accepted, we planned to proceed with the chi-square difference test to determine the best-fitting model.

We contrasted our hypothesized four-factor model against several alternative factor models. The results generated by AMOS 22.0 showed that the four-factor model provided a good model fit (χ^2^ = 1108.550, χ^2^/*df* = 4.121, GFI = 0.952, TLI = 0.903, CFI = 0.911, RMSEA = 0.077), suggesting that the discriminant validity of the study variables were fairly high (see Table 1). The standardized factor loading of each item from the latent constructs ranged from 0.53 to 0.80 and all were statistically significant (*p* < 0.001). Therefore, the convergent validity was verified.

**Table 1:** Comparison of the measurement models.

Factor models	χ^2^	*df*	χ^2^/*df*	GFI	TLI	CFI	RMSEA
Single-factor model	3488.673	275	12.686	0.543	0.430	0.478	0.170
Two-factor model	2612.634	274	9.535	0.689	0.584	0.620	0.146
Three-factor model	2566.993	272	9.437	0.690	0.589	0.627	0.145
Four-factor model	1108.550	269	4.121	0.952	0.903	0.911	0.077
CMF model	1070.571	244	4.388	0.960	0.905	0.917	0.077

*Single-factor: WO + CH + PD + SQ; two-factor: WO, CH + PD + SQ; three-factor: WO, CH, PD + SQ; four-factor: WO, CH, PD, SQ; WO, workplace ostracism; CH, coping humor; PD, psychological detachment; SQ, sleep quality; CMF, common method factor.*

Podsakoff et al. (2003) argue that CMB may exist when data are collected via self-report questionnaires. Such concerns may hamper us in drawing the correct causal relationships among the study variables (Li and Chen, 2018). To address this problem, we used two methods to check whether CMB existed in our research. First, Harman’s single-factor test was used, in which all items of our study variables were combined into one factor analysis, to preliminarily test for the existence of CMB. As expected, Harman’s single-factor test demonstrated that the variance explained by the first factor was 31.52%, far less than the 50% threshold indicative of problematic CMB (Hair et al., 1998). Then, a single common method factor (CMF) approach was also employed to further check for the existence of CMB through CFA (Boiral and Paillé, 2012; Ng et al., 2014). First, we built the CMF model, namely, the total 25 measurement items that were loaded on the underlying theoretical constructs and on a created latent construct labeled CMB separately. Then the model fit indices were compared between the CMF model and the four-factor model. The result indicated that the fit indices of the CMF model (χ^2^ = 1070.571, χ^2^/*df* = 4.388, GFI = 0.960, TLI = 0.905, CFI = 0.917, RMSEA = 0.077) all reached acceptable levels. However, the chi-square difference between the CMF model and the four-factor model [Δχ^2^(25) = 37.98, *p* > 0.05] was not significant, which suggested that the CMF model did not significantly improve model fit (see Table 1). Accordingly, the potential difficulty of CMB could be ignored in the present research.

### Descriptive Statistics

Table 2 displays descriptive statistics and the correlations between the study variables. As expected, the core study variables were significantly associated with each other. More specifically, workplace ostracism was negatively related to coping humor (*r* = −0.38, *p* < 0.001), psychological detachment (*r* = −0.49, *p* < 0.001) and sleep quality (*r* = −0.33, *p* < 0.001). Coping humor was positively correlated with psychological detachment (*r* = 0.55, *p* < 0.001) and sleep quality (*r* = 0.37, *p* < 0.001). In addition, psychological detachment was positively associated with sleep quality (*r* = 0.63, *p* < 0.001).

**Table 2:** Descriptive statistics and correlations among all variables.

Variables	1	2	3	4	5	6	7	8	*M*	*SD*
1. Gender	–								0.52	0.50
2. Age	0.158^∗∗^	–							34.03	6.74
3. Marital status	0.211^∗∗∗^	0.461^∗∗∗^	–						1.81	0.47
4. Daily working hours	0.283^∗∗∗^	0.055	0.209^∗∗∗^	–					10.44	2.42
5. Workplace ostracism	−0.152^∗∗^	0.050	−0.107^∗^	0.199^∗∗∗^	(0.89)				2.63	0.64
6. Coping humor	0.027	0.056	0.194^∗∗∗^	−0.111^∗^	−0.381^∗∗∗^	(0.83)			3.44	0.43
7. Psychological detachment	−0.006	−0.009	−0.084	−0.198^∗∗^	−0.491^∗∗∗^	0.554^∗∗∗^	(0.77)		3.39	0.75
8. Sleep quality	−0.010	−0.075	−0.009	−0.268^∗∗∗^	−0.330^∗∗∗^	0.373^∗∗∗^	0.626^∗∗∗^	(0.79)	2.97	0.85

*Gender: 0-female, 1-male; Marital status: 1-unmarried, 2-married, 3-others; Cronbach’s alphas are in parentheses on the diagonal. ^∗^p < 0.05, ^∗∗^p < 0.01, ^∗∗∗^p < 0.001.*

### Mediation and Moderation Testing

We applied the bootstrapping procedure (Preacher and Hayes, 2004) with 1,000 subsamples to estimate the direct and indirect effects in our conceptual model. Bootstrapping is more powerful than the method of Baron and Kenny (1986) to check the mediation effect, as it is unnecessary to assume that samples are of normal distribution and it also can obtain a more confident estimation of the indirect effect (Mackinnon et al., 2002; Chun et al., 2014; Chen et al., 2017). If the 95% confidence interval (CI) does not contain zero, then the direct and indirect effects are significant. Table 3 presents the results of the bootstrapping analysis after we controlled for the effects of gender, age, marital status, and daily working hours.

**Table 3:** The direct and indirect effects and 95% confidence intervals in the conceptual model.

Model paths	Estimated effect	95% CI	
		Lower bounds	Upper bounds
**Total effect**			
WO → SQ	−0.31	−0.418	−0.181
**Direct effect**			
WO → PD	−0.49	−0.567	−0.414
PD → SQ	0.59	0.518	0.670
WO → SQ	−0.02	−0.111	0.085
**Indirect effect**			
WO → PD → SQ	−0.29	−0.353	−0.229

*WO, workplace ostracism; PD, psychological detachment; SQ, sleep quality.*

As shown in Table 3, the results of the bootstrap sampling showed that workplace ostracism was negatively related to psychological detachment (β = −0.49, 95% CI = [−0.567, −0.414]), supporting Hypothesis 1. The direct effect of psychological detachment on sleep quality was significant (β = 0.59, 95% CI = [0.518, 0.670]), supporting Hypothesis 2. The indirect effect of workplace ostracism on sleep quality via psychological detachment reached a significant level (β = −0.29, 95% CI = [−0.353, −0.229]), thus Hypothesis 3 was supported. In addition, considering that the direct effect of workplace ostracism on sleep quality was non-significant (β = –0.02, 95% CI = [−0.111, 0.085]), psychological detachment fully mediated the relationship between workplace ostracism and sleep quality.

### Mediated Moderation Testing

To validate this mediated moderation relationship, we adopted the approach of Muller et al. (2005). According to this approach, we constructed several equations:

(1) $$Y=\beta_{10}+\beta_{11}X+\beta_{12}\mathrm{Mo}+\beta_{13}\mathrm{XMo}+\varepsilon_{1}$$

(2) $$\mathrm{Me}=\beta_{20}+\beta_{21}X+\beta_{22}\mathrm{Mo}+\beta_{23}\mathrm{XMo}+\varepsilon_{2}$$

(3) $$Y=\beta_{30}+\beta_{31}X+\beta_{32}\mathrm{Mo}+\beta_{33}\mathrm{XMo}+\beta_{34}\mathrm{Me}+\beta_{35}\mathrm{MeMo}+\varepsilon_{2}$$

In these three equations, *X*, *Mo*, *Me*, and *Y* represent the independent variable, moderator, mediator and dependent variables, respectively. There are some conditions that should be met for this relationship to be supported. First, in equation (1), β_13_ should be significant; Second, in Eqs. (2) and (3), either (or both) of two patterns should exist: both β_23_ and β_34_ are significant or both β_21_ and β_35_ are significant. Third, β_33_ should be smaller than β_13_ (or β_33_ is non-significant in the case of what might be called “full” mediated moderation). Given that we proposed that coping humor buffers the effects of workplace ostracism on sleep quality and psychological detachment, thus β_13_, β_23_, and β_34_ must be significant, and β_33_ should be smaller than β_13_ in this study. Table 4 provides the regression and bootstrap results for the mediated moderation model.

**Table 4:** Regression and bootstrap results for the moderation and mediated moderation models.

Variables	Equation (1)		Equation (2)		Equation (3)	
	SQ		PD		SQ	
	Model 1	Model 2	Model 3	Model 4	Model 5	Model 6
***Control variables***						
Gender	−0.079	−0.075	0.061	0.072	−0.079	−0.038
Age	−0.069	−0.090	0.022	0.036	−0.069	−0.072
Marital status	−0.023	−0.029	−0.065	−0.133^∗∗^	−0.023	−0.036
Daily working hours	−0.299^∗∗∗^	−0.335^∗∗∗^	−0.203^∗∗∗^	−0.237^∗∗∗^	−0.299^∗∗∗^	−0.222^∗∗∗^
***Independent variable (X)***						
WO		−0.096 (β_11_)		−0.208^∗∗∗^ (β_21_)		−0.013 (β_31_)
***Moderator (Mo)***						
CH		0.483^∗∗∗^ (β_12_)		0.643^∗∗∗^ (β_22_)		0.156^∗^ (β_32_)
***Interaction 1***						
WO × CH		0.233^∗∗∗^ (β_13_)		0.253^∗∗∗^ (β_23_)		0.121^∗^ (β_33_)
***Mediator (Me)***						
PD						0.509^∗∗∗^ (β_34_)
***Interaction 2***						
CH × PD						−0.031 (β_35_)
*R*^2^	0.086	0.304	0.045	0.501	0.086	0.437
Δ*R*^2^	0.086^∗∗∗^	0.218^∗∗∗^	0.045^∗∗^	0.456^∗∗∗^	0.086^∗∗∗^	0.351^∗∗∗^

**Model paths**	**Coping humor**		**Estimated effect**		**95% CI**	

WO → PD → SQ	High (+1 SD)		−0.059		[−0.127, 0.003]	
	Low (−1 SD)		−0.248		[−0.318, −0.181]	

*WO, workplace ostracism; PD, psychological detachment; CH, coping humor; SQ, sleep quality. ^∗^p < 0.05, ^∗∗^p < 0.01, ^∗∗∗^p < 0.001.*

We used hierarchical multiple regression analysis to check the mediated moderation model. To remove the interference of multicollinearity to the results, workplace ostracism, coping humor, and psychological detachment were mean centered (Muller et al., 2005; Dalal and Zickar, 2012; Jose, 2013). First, sleep quality and psychological detachment were regressed on the control variables, workplace ostracism, coping humor, and interaction term 1 (i.e., WO × CH), respectively (Model 2 and Model 4, Table 4). Then, sleep quality was regressed on the control variables, workplace ostracism, coping humor, interaction term 1 (i.e., WO × CH), psychological detachment, and interaction term 2 (i.e., CH × PD). As can be seen in Table 4, the interaction term 1 (WO × CH) was significantly and positively related to psychological detachment (β = 0.253, *p* < 0.001; Model 4), which indicated that coping humor plays a moderating role in the relationship between workplace ostracism and psychological detachment. At the same time, the coefficient of the interaction term 1 (WO × CH) was also significant (β = 0.233, p < 0.001) in Model 2. Therefore, the link between workplace ostracism and sleep quality was also moderated by coping humor.

**FIGURE 2:**
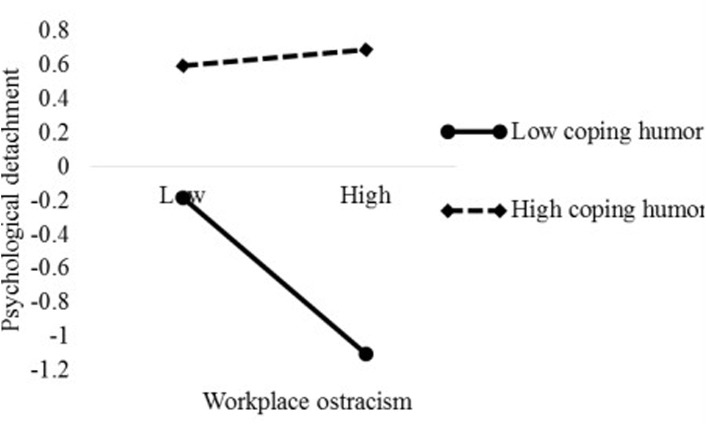
The moderation effect of coping humor on the relationship between workplace ostracism and psychological detachment.

**FIGURE 3:**
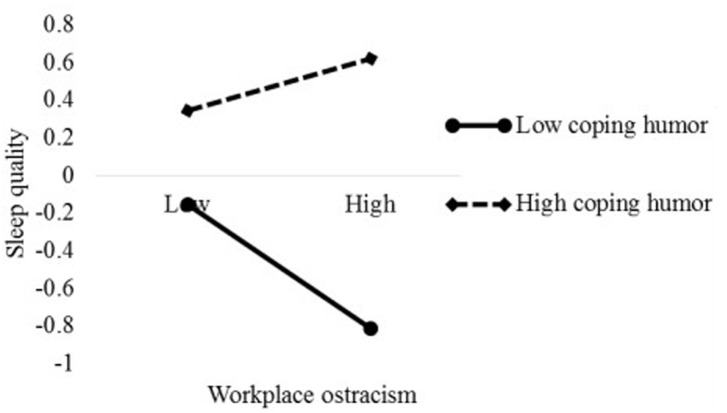
The moderation effect of coping humor on the relationship between workplace ostracism and sleep quality.

To better interpret the moderation patterns of coping humor, we used the procedure of Aiken and West (1994), namely one *SD* above (high level) and below (low level) the mean of coping humor were selected to compute and plot the slopes. As demonstrated in Figure 2, we found that workplace ostracism had a significant effect on psychological detachment when coping humor was low (simple slope = −0.461, *p* < 0.001) but no significant relationship was found in for high coping humor (simple slope = 0.045, *p* > 0.05). Therefore, Hypothesis 4 was supported. Similarly, Figure 3 shows that workplace ostracism was more negatively related to sleep quality for low coping humor (simple slope = −0.329, *p* < 0.001) than high coping humor (simple slope = 0.137, *p* < 0.05), supporting Hypothesis 5.

A review of Table 4 suggests that β_13_, β_23_, and β_34_ all reached significant levels, and β_33_ was smaller than β_13._ The indirect effect of workplace ostracism on sleep quality through psychological detachment was significant but negative when coping humor was low (β = −0.248, 95% CI = [−0.318, −0.181]). However, the indirect effect became insignificant with high coping humor (β = −0.059, 95% CI = [−0.127, 0.003]). Accordingly, Hypothesis 6 was supported.

## Discussion

By drawing on the ESD model, the central aims of this study were to reveal the underlying mechanisms and boundary conditions for the link between workplace ostracism and sleep quality. First, we demonstrated that workplace ostracism was negatively related to psychological detachment, which is in keeping with the previous research findings of Chen and Li (2018), and suggests that the negative effect of workplace ostracism can spill over to non-work domains (Liu et al., 2013). As an interpersonal stressor from social interaction, workplace ostracism can lead employees experiencing prolonged negative activation and the hampering of their psychological detachment. This is congruent with Sonnentag and Fritz (2015) assumption that job stressors, including social stressors, are important antecedents of insufficient psychological detachment. Moreover, we confirmed that poor psychological detachment was associated with low sleep quality, which to some extent reflects that psychological detachment is a prototypical recovery experience supporting recovery processes (Sonnentag and Fritz, 2007). This finding is in line with that of Clinton et al. (2017), in which psychological detachment was positively related to sleep quality.

Second, we proposed that psychological detachment mediates the relationship between workplace ostracism and sleep quality. The findings confirm that psychological detachment plays a fully mediating role in the relationship between workplace ostracism and sleep quality. More specifically, the detrimental effects of workplace ostracism can spill over to employees’ non-work domains and trigger their negative activation (e.g., rumination, worry, and negative affect). This obstructs psychological detachment at bedtime, thereby impairing sleep quality. This result is similar to the findings from Berset et al. (2011) research which indicated that rumination (one kind of insufficient psychological detachment) mediated the relationship between workplace stressors and sleep quality. Furthermore, according to the temporal need-threat model of Williams (2009), this result also suggests that we may be assessing the resignation stage of workplace ostracism which means that as individuals long-term exposure to ostracism events, the resources which can fortify the threatened needs are depleted over time. In this case, individuals have difficulty in recovering from ostracism, and then bring about undesirable outcomes (Williams, 2009). The current result–namely, workplace ostracism being assessed over a 1-month period influences employees’ psychological detachment and sleep quality, which is in line with the description of the resignation stage of ostracism. It should be noted that Pereira et al. (2013) explored the mediating mechanism between social exclusion at work and sleep quality using worries about work as a mediator. However, the results suggested that worries about work did not play a mediating role. Although there are some overlaps between worry and lack of detachment, lack of detachment is not identical to worry, and psychological detachment is not simply the opposite of worry (Sonnentag and Fritz, 2015). From the definition of psychological detachment, we are aware that if individuals engage in work-related activities, thoughts or worries during non-work time, then they are suffering from a lack of psychological detachment. Worry is just one form of a lack of psychological detachment and the scope of psychological detachment is much broader than worries about work during off-hours. Empirical evidence also supports that lack of detachment is not identical with worry (e.g., Flaxman et al., 2012). In addition, the questionnaire of social exclusion at work adopted by Pereira et al. (2013) is less effective than Workplace Ostracism Scale of Ferris et al. (2008b). These may be the reasons why the mediating effect was supported in our study but not in the study by Pereira et al. (2013).

Third, we found that coping humor tended to act as a buffer in the relationships between workplace ostracism and psychological detachment, and sleep quality. That is, people who have high levels of coping humor tend to generally experience a lessened poor psychological detachment and sleep quality due to workplace ostracism than their low coping humor counterparts. These results appear to mirror those from Martin and Dobbin (1988) research, which suggests that humor has a stress-moderating effect. These results also are broadly in line with the research findings of Sliter et al. (2014), in which coping humor was found to weaken the effects of traumatic stressors on individual outcomes of firefighters.

Fourth, the association between job stressors and sleep problems are more complex than we expected (Knudsen et al., 2007). With this idea in mind and by integrating mediation and moderation, we developed a model of mediated moderation in which psychological detachment mediated the moderation effect of coping humor on the relationship between workplace ostracism and sleep quality. The result mainly supported the notion that compared with high coping humor, employees with low coping humor may further exacerbate the negative effects of workplace ostracism on sleep quality via insufficient psychological detachment. This finding is significant in the methodology of revealing the mechanism between workplace ostracism and sleep quality by adopting the mediated moderating model.

### Theoretical Implications

The findings of the present study suggest some theoretical implications. First and foremost, we revealed the internal mechanism between workplace ostracism and sleep quality based on the ESD model rather than the Need to Belong Theory (Baumeister and Leary, 1995). Despite previous studies (e.g., Pereira et al., 2013; Pereira and Elfering, 2014a) exploring the relationship between social exclusion/social stressors at work and sleep quality by drawing on the Need to Belong Theory, we argued that sleep is one of the most important recovery processes, and that it should be put into the framework of recovery to reveal its formulation mechanism. In this study, we identified a complex relationship between workplace ostracism and sleep quality based on the ESD model which is a key theoretical model in the field of recovery (Chen and Li, 2018). Although the ESD model did not mention mediated moderation relationships, we found that psychological detachment mediates the moderation effect of coping humor on the relationship between workplace ostracism and sleep quality. As such, the present study advances not only the ESD model but also offers a distinct perspective to explain how and when workplace ostracism affects sleep quality.

Second, this study sheds light on psychological detachment as an intervening mechanism which accounts for the relationship between workplace ostracism and sleep quality. By including psychological detachment as a new mediator in the relationship between workplace ostracism and sleep quality, we extend the previous research of Pereira et al. (2013) which focused on the role of worries about work. In addition, we also responded to the suggestion from Ferris et al. (2008b) to test the mediating mechanisms between workplace ostracism and employees’ outcomes.

Third, by introducing coping humor as a moderator in the relationships between workplace ostracism and psychological detachment, and sleep quality, this study not only enriches the research regarding coping humor but also addresses the limitation that previous research has not considered the boundary conditions when testing the relationship between social stressors at work and sleep (e.g., Pereira et al., 2013, 2016; Pereira and Elfering, 2014a,b), and deepens our understanding of the relationship between workplace ostracism and sleep quality. Furthermore, it provides empirical evidence for the argument that workplace ostracism may be extremely detrimental only to some people but not everyone (Williams and Sommer, 1997; Ferris et al., 2008b).

Last but not least, we explored the detrimental effect of workplace ostracism on sleep quality by investigating a sample of 403 employees in China. Pereira et al. (2013) demonstrated this relationship to some extent through their study of 90 full-time employees from Switzerland. Therefore, our findings expand understanding of the cross-cultural generalizability of the association between workplace ostracism and sleep quality.

### Practical Implications

Our findings also suggest some practical implications for managers and policymakers. First, as our findings show that workplace ostracism is negatively associated with psychological detachment and sleep quality, managers/policymakers should take some measures to prevent or eliminate the detrimental effects of workplace ostracism. Several approaches might help to achieve this aim. Organizations can foster a harmonious and inclusive climate. For instance, employees are encouraged to express their voices based on mutual respect and frequently take part in group activities that consequently enhance interpersonal relationships (Riva and Eck, 2016). Moreover, when group members are assigned common tasks and/or the reward systems are changed from being individual-oriented to team-oriented, then employees are more likely to cooperate with others and workplace ostracism can be discouraged (Wu et al., 2015). In addition, employees with the personality characteristics of neuroticism and introversion are more likely to perceive workplace ostracism (Wu et al., 2011). Thus, during the recruitment process, the Big Five Questionnaire (Costa and McCrae, 1987) should be used to identify neurotic and introverted individuals and avoid hiring them.

Second, considering that psychological detachment has positive impacts on sleep quality, both employees and organizations can adopt some interventions to strengthen psychological detachment. More specifically, to effectively detach from work, employees need to engage in some activities they are interested in after leaving the work environment. For example, they can undertake volunteer work and leisure activities, such as taking vacations and going to parties, rather than thinking about work-related issues during their non-work time (Etzion et al., 1998; Sonnentag, 2012). Moreover, some physical and/or psychological boundaries can be set between work and non-work domains to enhance employees’ psychological detachment (Ashforth et al., 2000). These include checking work-related emails only at work, avoiding work-related phone calls after work, and not talking about work-related issues at the end of the workday (Sonnentag and Fritz, 2015). As for organizations, supervisors should be discouraged from assigning tasks to employees during non-work time unless in the case of emergencies. Furthermore, organizations should provide some training and guidance to employees with insufficient psychological detachment, such as in setting priorities, time-management skills, and job skills, to ensure they finish tasks on time and avoid ruminating on work-related issues in off-hours (Sonnentag et al., 2014; Chen et al., 2017).

Finally, the impact of humor should be considered. As suggested by our findings, coping humor is a useful way to deal with workplace ostracism and undermine its negative effects on psychological detachment and sleep quality. Therefore, some measures should be taken to improve employees’ humor. Organizations can adopt employee training and humor management. For example, to strengthen the ability of employees to use humor as a way of coping with occupational stressors in their daily life, trainers can provide humor training courses and play humorous videos. In addition, employees should be encouraged to share some funny jokes. In these ways, employees will restructure their cognitions and reevaluate the situation, making it seem less stressful (Sliter et al., 2014).

### Limitations and Future Research

Although the present study has some theoretical and practical implications, the limitations should also be considered. First, Chinese employees were used as the research sample, which may restrict the findings of the present study from being generalized to other cultures or countries. In Chinese society, individuals tend to establish and maintain harmonious interpersonal relationships with their colleagues, and they are more vulnerable to negative issues under the influence of collectivism which is a dominant culture in China (Liu et al., 2009; Powell et al., 2009). Therefore, the negative effects of workplace ostracism may be augmented in Chinese society (Wu et al., 2012). In addition, due to the influences of collectivism and individualism, peoples from the East (e.g., Chinese and Japanese) have high interdependent self-construal, while those from the West (e.g., Europeans and Americans) have low one (Cross et al., 2011), and peoples with high levels of interdependent self-construal are recovery from the ostracism episode more quickly than those with low levels (Ren et al., 2013). Thus the effect of workplace ostracism on employees in the Chinese context may differ from that in other countries/societies (Zhu et al., 2017). Future research should examine our conceptual model in different countries with distinct dominant cultures.

Second, all our study variables were self-rated by employees and this method is prone to social desirability effects in responding and the “face” culture. For example, when employees assess their perceived workplace ostracism, they may under-report the real perception of workplace ostracism to avoid being identified as being unpopular. Moreover, due to the fact that our data were collected in two separate stages, the influence of the original ostracism events in T1 could have decreased over that 1 month period, which may cause the changes of causality. In addition, we did not have control over such as pre-sleep arousal (C̀apková et al., 2018), sleep hygiene (Markus et al., 2018), dysfunctional beliefs about sleep (Eidelman et al., 2016) and alcohol use (Chakravorty et al., 2016), that are known to affect sleep quality that could have had an influence on the findings of this study. Thus, in future research, we recommend that (1) some other kinds of measures (e.g., coworker-reports and supervisor-reports) should be employed to assess our study variables; (2) workplace ostracism should be measured once again in T2 to confirm whether it is different from that in T1; (3) some other factors which affect sleep quality can be controlled for in our concept model to obtain more precise findings.

Third, the sources of workplace ostracism were not differentiated (e.g., ostracism from supervisors and coworkers). Researchers argue that different sources of workplace ostracism may generate distinct effects and may interact to impact the individuals’ outcomes (Williams, 2007; Ferris et al., 2008b; Zhu et al., 2017). It is not clear whether the same conclusions would be gained if we distinguish between different sources of workplace ostracism. For this, future researchers are encouraged to test the impacts of different sources of workplace ostracism on employees’ psychological detachment and sleep quality.

## Conclusion

The relationship between workplace ostracism and sleep quality turned out to be more complex than it initially seemed. Some evidence previously suggested that workplace ostracism affects sleep quality in a negative way. However, empirical research about how and when workplace ostracism impacts on sleep quality is limited. The current study revealed that compared with high coping humor counterparts, employees with low coping humor are more likely to be affected by workplace ostracism and experience lower sleep quality via decreased psychological detachment. These findings should be of benefit to organizational research on workplace ostracism and the ESD model, and help organizations to redesign human resources policies.

## Ethics Statement

This study was carried out in accordance with the recommendations of ethics committee of China University of Mining and Technology with written informed consent from all subjects. All subjects gave written informed consent in accordance with the Declaration of Helsinki. The protocol was approved by the ethics committee of China University of Mining and Technology.

## Author Contributions

YC designed and drafted the work. SL collected and analyzed the data for the study.

## Conflict of Interest Statement

The authors declare that the research was conducted in the absence of any commercial or financial relationships that could be construed as a potential conflict of interest.
